# Massive pericardial effusion and cardiac tamponade revealed undiagnosed Turner syndrome: a case report

**DOI:** 10.1186/s12872-020-01728-2

**Published:** 2020-10-23

**Authors:** Wei Qiang, Rongxin Sun, Xiaopu Zheng, Yuan Du

**Affiliations:** 1 Department of Endocrinology, The First Affiliated Hospital of Xi’an Jiaotong University, Xi’an, 710061 People’s Republic of China; 2 Beijing Key Laboratory of Diabetes Research and Care, Center for Endocrine Metabolism and Immune Diseases, Luhe Hospital, Capital Medical University, Beijing, 101149 People’s Republic of China; 3 Department of Cardiovascular Medicine, The First Affiliated Hospital of Xi’an Jiaotong University, No. 277 West Yanta Road, Xi’an, 710061 People’s Republic of China

**Keywords:** Pericardial effusion, Cardiac tamponade, Autoimmune thyroid disease, Hypothyroidism, Turner syndrome

## Abstract

**Background:**

Patients with Turner syndrome (TS) are prone to autoimmune disorders. Although most patients with TS are diagnosed at younger ages, delayed diagnosis is not rare.

**Case presentation:**

A 31-year-old woman was presented with facial edema, chest tightness and dyspnea. She had primary amenorrhea. Physical examination revealed short stature, dry skin and coarse hair. Periorbital edema with puffy eyelids were also noticed with mild goiter. Bilateral cardiac enlargement, distant heart sounds and pulsus paradoxus, in combination with hepatomegaly and jugular venous distention were observed. Her hircus and pubic hair was absent. The development of her breast was at 1st tanner period and gynecological examination revealed infantile vulva. Echocardiography suggested massive pericardial effusion. She was diagnosed with cardiac tamponade based on low systolic pressure, decreased pulse pressure and pulsus paradoxus. Pericardiocentesis was performed. Thyroid function test and thyroid ultrasound indicated Hashimoto’s thyroiditis and severe hypothyroidism. Sex hormone test revealed hypergonadotropin hypogonadism. Further karyotyping revealed a karyotype of 45, X [21]/46, X, i(X) (q10) [29] and she was diagnosed with mosaic + variant type of TS. L-T4 supplement, estrogen therapy, and antiosteoporosis treatment was initiated. Euthyroidism and complete resolution of the pericardial effusion was obtained within 2 months.

**Conclusion:**

Hypothyroidism should be considered in the patients with pericardial effusion. The association between autoimmune thyroid diseases and TS should be kept in mind. Both congenital and acquired cardiovascular diseases should be screened in patients with TS.

## Background

Turner syndrome (TS) is a rare condition with short stature, ovarian dysgenesis and infertility in women as the result of either complete or partial loss of one X chromosome. TS is also associated congenital heart malformations, metabolism disorders such as diabetes mellitus and osteoporosis. Patients with TS are prone to autoimmune disorders among which Hashimoto’s thyroiditis is the most common one [1]. The average age at diagnosis is around 15 years old, but delayed diagnosis is not rare [2]. Here, we reported woman diagnosed with TS at the age of 31, with initial presentation of massive pericardial effusion and cardiac tamponade due to very severe hypothyroidism. This case emphasized the association between thyroid diseases and TS and also the necessity of a multidisciplinary approach to care.

## Case presentation

**Fig. 1:**
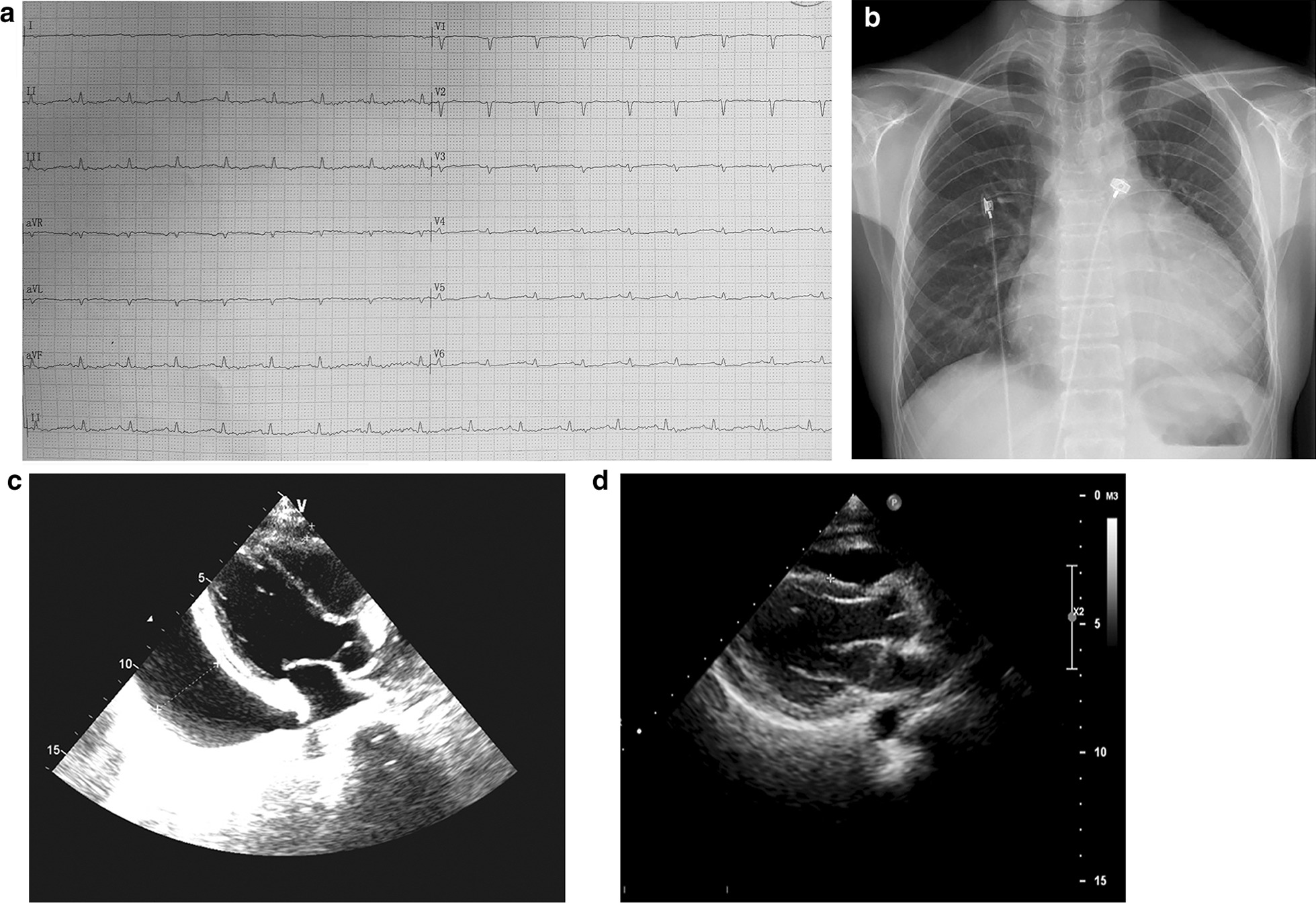
Cardiovascular findings of the patient at initial admission. **a** ECG showed low voltage in all precordial leads. **b** Chest X-ray suggested cardiomegaly. **c** Transthoracic echocardiography indicated massive pericardial effusion. **d** Resolution of the pericardial effusion

A 31-year-old woman was presented at cardiovascular department with facial edema for 2 months and chest tightness for 1 week. Fatigue, palpitation and exertional dyspnea were also reported. She had primary amenorrhea. The patient’s vital signs were: T 36.1℃, P 110 bpm, R 20 bpm, BP 80/70 mmHg. She was 141 cm high and weighed 41 kg. Physical examination revealed short stature, dry skin and coarse hair. Periorbital edema with puffy eyelids were also noticed with mild goiter. Bilateral cardiac enlargement, distant heart sounds and pulsus paradoxus, in combination with hepatomegaly and jugular venous distention were observed. There was no pretibial non-pitting edema. Her hircus and pubic hair was absent. The development of her breast was at 1st Tanner period and gynecological examination revealed infantile vulva. The electrocardiogram (ECG) showed low voltage in all leads (Fig. 1a) and the chest X-Ray showed cardiomegaly (Fig. 1b). Massive pericardial effusion was the most protruding finding on echocardiography (33 mm behind the posterior wall of the left ventricle, 6 mm in front of the anterior wall of the right ventricle, 23 mm outside the lateral wall of the left ventricle) (Fig. 1c). The size and structure of the heart were normal and the left ventricular ejection fraction was 63%. Elevated liver enzymes were reported during routine evaluation (Table 1). She was diagnosed with cardiac tamponade based on low systolic pressure, decreased pulse pressure and pulsus paradoxus. Pericardiocentesis was performed and her dyspnea was alleviated after the drainage of pericardial effusion (Additional file 1: Fig. S1). No increases of white blood cell (WBC), percentage of neutrophil or procalcitonin were found (Table 1). Some etiologies for pericardial effusion including tuberculosis, connective tissue diseases and tumors were excluded by measurement of antibodies and biomarkers (Additional file 2: Table S1). Acid-fast staining, bacterial and fungal cultures of the effusion fluid were also negative.

**Table 1:** Laboratory findings on admission of the patient

Parameters	Patient’s values	Reference range
BMI	20.62	18–24 kg/m^2^
Blood pressure		
SBP	80	≤ 120 mmHg
DBP	70	≤ 80 mmHg
Glucose metabolism		
HbA1c	5.2	4.0–6.0%
Glucose (0 h)	4.30	3.9–6.1 mmol/L
Glucose (2 h)	4.63	< 7.8 mmol/L
Insulin (0 h)	11.3	5.0–25.0 mIU/L
Insulin (2 h)	50.6	
Lipid		
Total CHO	5.33	3.1–5.69 mmol/L
TG	1.22	0.56–1.47 mmol/L
LDL	3.13	2.07–3.10 mmol/L
HDL	1.78	1.29–1.55 mmol/L
Uric acid	293	155–357 μmol/L
Blood routine		
RBC	3.44	3.8–5.1 * 10^12^/L
HGB	130	110–150 g/L
WBC	5.4	3.5–9.5 * 10^9^/L
NEUT%	71.8	40–75%
Liver function		
ALT	37	7–40 IU/L
AST	62	13–35 IU/L
ALP	196	35–100 IU/L
GGT	239	7–45 IU/L
Renal function		
BUN	5.4	2.6–7.5 mmol/L
CRE	75	41–81 μmol/L
Electrolytes		
Na	138	137–147 mmol/L
K	3.46	3.5–5.3 mmol/L
Cl	85.6	96–108 mmol/L
Hydropericardium		
CHO	2.20	3.1–5.69 mmol/L
Rivalta reaction	Positive	Negative

BMI, body mass index; SBP, systolic blood pressure; DBP, diastolic blood pressure; HbA1c, hemoglobin A1c; CHO, cholesterol; TG, triglyceride; LDL, low density lipoprotein; HDL, high density lipoprotein; RBC, red blood cell; HGB, hemoglobin; WBC, white blood cell; NEUT%, neutrophil ratio; ALT, alanine aminotransferase; AST, aspartate aminotransferase; ALP, alkaline phosphatase; GGT, γ-Glutamyl transferase; BUN, blood urea nitrogen; CRE, creatinine; Na, sodium; K, potassium; Cl, chloride

Thyroid function indicated severe hypothyroidism with positive TPOAb (Table 2). Further thyroid ultrasound revealed diffuse inhomogeneous hypo-echoic area. The diagnosis of Hashimoto thyroiditis was made. In view of primary amenorrhea, short stature and the absence of the secondary sexual characteristics, Turner syndrome (TS) was under suspicion. Pelvic ultrasound and sex hormone test were arranged, and absent ovaries, infantile uterus, decreased estrogen and elevated follicle stimulating hormone (FSH) were reported. Further karyotyping of peripheral blood cells revealed a karyotype of 45, X/46, X, i(X) (q10). The patient was diagnosed with mosaic and variant type of TS. Functions of other endocrine glands were tested and no abnormalities were found. Metabolism of glucose, lipid and uric acid (UA) were also evaluated (Table 1). The bone mineral density (BMD) was assessed and the T value of femur and lumbar vertebra (L1 to L4) were -3.5 and -3.6, respectively. Sensorineural deafness was revealed by electrical audition test.

**Table 2:** Function of endocrine glands

Parameters	Patient’s values	Reference range
Thyroid function on admission		
TPOAb^a^	> 3000	< 15 U/mL
TSH^a^	> 100	0.25–5 μIU/mL
T3^a^	< 0.05	0.78–2.20 ng/mL
T4^a^	< 2.00	4.2–13.5 μg/dL
Free T3^a^	2.94	2.91–9.08 pmol/L
Free T4^a^	3.00	9.05–25.5 pmol/L
TSH^b^	12.1	0.25–5 μIU/mL
Free T3^b^	4.79	2.91–9.08 pmol/L
Free T4^b^	19.2	9.05–25.5 pmol/L
Pituitary–gonadal axis		
E2	< 18.4	28–156 pmol/L
Prog	2.00	0.7–4.3 nmol/L
PRL	12.84	4.79–23.3 ng/mL
LH	6.28	1.7–8.6 mIU/mL
FSH	27.32	1.5–12.4 mIU/mL
T	< 0.087	0.29–1.67 nmol/L
Pituitary-adrenal axis		
ACTH	37.9	7.2–63.3 pg/mL
COR (8 am)	30.8	5–28 μg/dL
GH/IGF-1		
GH	< 1.7	< 10 μg/L
IGF-1	169	115–307 ng/mL
IGF-BP3	4.23	3.5–7.0 μg/mL

TPOAb, thyroid peroxidase antibody; TSH, thyroid-stimulating hormone; T3, triiodothyronine; T4, thyroxine; E2, estradiol; Prog, progesterone; PRL, prolactin; LH, luteinizing hormone; FSH, follicle-stimulating hormone; T, testosterone; ACTH, adrenocorticotropic hormone; COR, cortisol; GH, growth hormone; IGF-1, insulin-like growth factor 1; IGF-BP3, insulin-like growth factor binding protein 3

^a^Throid function at presentation

^b^TSH, FT4 and FT3 after one month of L-T4 sumplement

Levothyroxine (L-T4) supplement was initiated (25 μg/d for 3 days, 50 μg/d for 1 week and then 75 μg/d). Estrogen therapy and antiosteoporosis treatment were applied. The TSH was 12.1 μIU/mL with a normal FT4 after one month (Table 2). Meanwhile, resolution of the pericardial effusion was observed on echocardiography (Fig. 1d). L-T4 was increased to 112.5 μg/d and euthyroidism was reported one month later on the telephone follow-up.

## Discussion and conclusion

Here, we reported woman diagnosed with TS at the age of 31, with initial presentation of massive pericardial effusion and cardiac tamponade due to very severe hypothyroidism. The first difficulty in the diagnosis of this case is to identify that the cause of pericardial effusion is hypothyroidism induced by Hashimoto’s thyroiditis. The etiologies of pericardial effusions are numerous among which hypothyroidism is an uncommon one. The prevalence of pericardial effusion is 3–6% in patients with mild disease and 80% in myxedema [3–5]. It’s revealed during cardiovascular evaluation in most cases, but can also be the initial presentation. Increased capillary permeability and reduced lymphatic drainage result in the gradual accumulation of fluid in the pericardial space, though the mechanism hasn’t been fully elucidated [6]. The process usually takes from months to years, as the severity and chronicity of hypothyroidism varies [5, 7]. Moderate and massive effusions are rare [3, 4]. Pericardiocentesis is not necessary in most cases as pericardial effusion disappear within months on L-T4 treatment, with the exception of cardiac tamponade or for differential diagnosis.

The more challenging step was to reveal the underlying TS in this patient after a primary diagnosis of Hashimoto’s thyroiditis and hypothyroidism. This is not only based on her primary amenorrhea and abnormal physical examination, but also based on the alert of increased frequency of Hashimoto’s thyroiditis in patients with TS. As the X chromosome contains several immune-related genes [8], the loss of a distinct X chromosome locus could facilitate the development of autoimmune thyroid diseases (AITDs) [9]. As a result, patients with TS have the tendency to develop various autoimmune diseases, including AITDs, type 1 diabetes and inflammatory bowel disease (IBD), among which Hashimoto’s thyroiditis is the most common one [1]. F. Mortensen et al. and Chen et al. reported the increased frequency of AITDs with age [10, 11]. Besides, though euthyroidism is a common clinical phenotype, transition to subclinical or overt hypothyroidism can occur [12], emphasizing the importance of screening for thyroid diseases through life-span [13]. A higher prevalence of AITDs was reported in iso-chromosome Xq population [14, 15]. Further studies are required to elucidate the underlying mechanism. Despite the well documented role of estrogen in AITDs, no association between estrogen administration and the occurrence of AIDs was revealed in TS patients [9].

Another noteworthy issue is the screening for cardiovascular abnormalities of TS itself. Both congenital heart malformation and acquired disorder such as ischemic heart disease contributes greatly to morbidity and mortality in patients with TS. Bicuspid aortic valve is the most common congenital malformation with a prevalence of 14–34%. Other common phenotypic characteristics of heart include coarctation of the aorta (7–14%) and aortic dilation/aneurysm (3–42%) [2]. These cardiovascular malformations can occur in isolation or in combination in patients with TS [16–19]. Hypertension occurs in 50% of patients with TS [2]. Persistent elevation of systolic blood pressure is a risk factor for aortic root dilation and the consequent aortic dissection. The exact genetic and epigenetic mechanism of cardiopathies in TS remains unclear. Acquired heart abnormalities are associated with obesity, hyperlipidemia, hypertension, diabetes mellitus and low estrogen levels. Cardiovascular diseases, both congenital and acquired ones, should be screened in patients with TS.

In conclusion, this case indicated that hypothyroidism should be screened in patients with pericardial effusion, and also emphasized the link between autoimmune thyroid diseases and TS.

## Supplementary information

**Additional file 1: Fig. S1**. The process of pericardiocentesis under the guidance of ultrasound. a Confirmation of the puncture site. b–d Paracentetic needle in the pericardial cavity.

**Additional file 2: Table S1**. Screening results for other etiologies of pericardial effusion.

## Data Availability

All relevant data supporting the conclusions of this article are included within the article.

## Abbreviations

AITDs: Autoimmune thyroid diseases
ECG: Electrocardiogram
IBD: Inflammatory bowel disease
TS: Turner syndrome
