# Supplementary figures and images for: Massive pericardial effusion and cardiac tamponade revealed undiagnosed Turner syndrome: a case report
Source: BMC Cardiovasc Disord. 2020 Oct 23;20:459. doi: 10.1186/s12872-020-01728-2 (PMC7583196; doi:10.1186/s12872-020-01728-2)

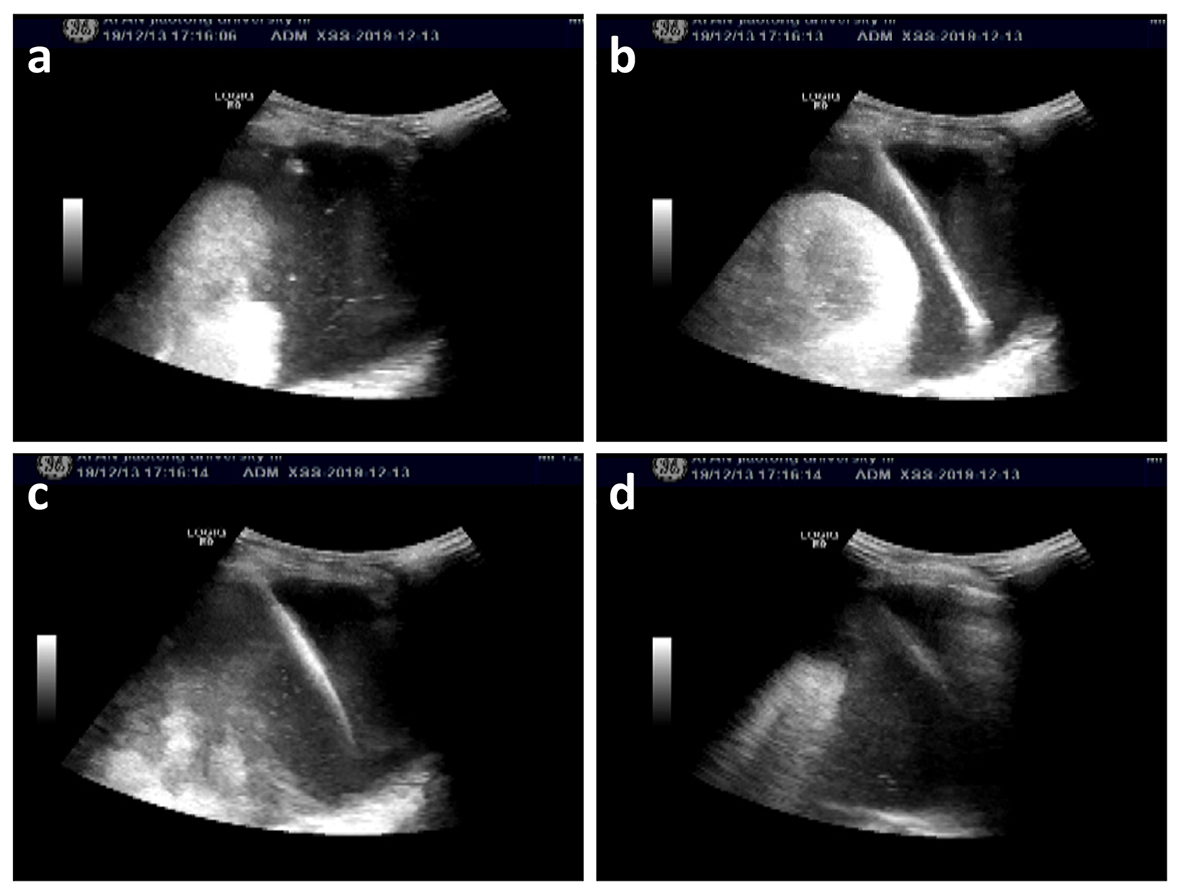

Supplement: Supplementary file 1 — Additional file 1: Fig. S1. The process of pericardiocentesis under the guidance of ultrasound. a Confirmation of the puncture site. b–d Paracentetic needle in the pericardial cavity. [file 12872_2020_1728_MOESM1_ESM.tif]
