# Supplementary material for: Massive pericardial effusion and cardiac tamponade revealed undiagnosed Turner syndrome: a case report
Source: BMC Cardiovasc Disord. 2020 Oct 23;20:459. doi: 10.1186/s12872-020-01728-2 (PMC7583196; doi:10.1186/s12872-020-01728-2)
Supplement: Supplementary file 2 — Additional file 2: Table S1. Screening results for other etiologies of pericardial effusion. [file 12872_2020_1728_MOESM2_ESM.docx]

| **Parameters** | **Patient’s values** | **Reference range** |
| --- | --- | --- |
| Connective tissue diseases |  |  |
| ANA | negative | negative |
| Ku | negative | negative |
| Mi-2 | negative | negative |
| Pm-Scl | negative | negative |
| Jo-1 | negative | negative |
| AMA M2 | negative | negative |
| U1-snRNP | negative | negative |
| Scl 70 | negative | negative |
| CENP-8 | negative | negative |
| SS-B/La | negative | negative |
| SSA/Ro 52kd | negative | negative |
| SSA/Ro 60kd | negative | negative |
| Rib Po | negative | negative |
| PCNA | negative | negative |
| SmD1 | negative | negative |
| Histones | negative | negative |
| Nucleosomes | negative | negative |
| dsDNA | negative | negative |
| Immunoassay |  |  |
| CRP | 0.48 | 0-3 mg/L |
| C4 | 0.21 | 0.1-0.4 g/L |
| C3 | 1.2 | 0.8-1.85 g/L |
| IgA | 3.54 | 0.7-3.8 g/L |
| IgM | 1.05 | 0.4-2.3 g/L |
| IgG | 9.3 | 7-16 g/L |
| IgE | 30 | 0-100 IU/mL |
| LAM light chain | 1.62 | 0.9-2.1 g/L |
| KAP light chain | 2.73 | 1.7-3.7 g/L |
| Tuberculosis (TB) |  |  |
| Mycobacterium TB antibody IgG | negative | negative |
| Mantoux TB test | negative | negative |
| Erythrocyte sedimentation rate | 13 | 0-20 mm/h |
| Tumor |  |  |
| CEA | 20.1 | 0-5 ng/mL |
| AFP | 14.7 | 0-7 ng/mL |
| CA125* | 175 | 0-35 U/mL |
| CA199 | 26.87 | 0-39 U/mL |
| CA724 | 0.54 | 0-6.9 U/mL |
| CA153 | 14 | 0-25 U/mL |

**Table S1. Screening results for other etiologies of pericardial effusion**

ANA, antinuclear antibodies; Ku, anti-Ku antibodies; Mi-2, anti-Mi-2 antibodies; Pm-Scl, anti-scleroderma antibodies; Jo-1, anti-Jo 1 antibodies; AMA M2, anti-mitochondrial M2 antibody; U1-snRNP, anti-U1 picribonucleoprotein antibody; Scl 70, anti-Scl 70 antibody; CENP-8, anti-centromere antibodies; SS-B/La, anti-SSB antibody; SSA/Ro 52kd, anti- SSA/Ro 52kd antibody; SSA/Ro 60kd, anti- SSA/Ro 60kd antibody; Rib Po, anti-ribosomal P protein antibody; PCNA, anti-proliferative cell antigen antibody; SmD1, anti-SmD1 antibodies; Histones, anti-histone antibody; Nucleosomes, anti-nucleosome antibodies; dsDNA, anti-double stranded DNA antibody; CRP, C-reactive protein; C4, complement 4; C3, complement 3; IgA, Immunoglobulin A; IgM, Immunoglobulin M; IgG, Immunoglobulin G; IgE, Immunoglobulin E; CEA, carcino-embryonic antigen; AFP, alpha fetal protein; CA125, carbohydrate antigen 125; CA 199, carbohydrate antigen 199; CA724, carbohydrate antigen 724; CA153, carbohydrate antigen 153.

*CA125 can be used as biomarker for ovarian cancer. However, it’s not specific and its elevation can occur in many noncancerous conditions including connective tissue diseases, chronic constrictive pericarditis, and pleural effusion [1.2]. In this case, as the patient was absent of ovaries, the elevation of CA125 is attributed to the massive pericardial effusion.

1. M Funauchi, S Ikoma, H Yu, M Sugiyama, M Ohno, K Kinoshita, et al. A case of progressive systemic sclerosis complicated by massive pleural effusion with elevated CA125. Lupus. 2000;9(5):382-5.
2. S F Hussain, J Grayez, A Grigorian, J T Green. Massive pleural effusion and marked increase of CA-125. Postgrad Med J. 2004;80(943):300-1.
